# Liquid biopsy in T-cell lymphoma: biomarker detection techniques and clinical application

**DOI:** 10.1186/s12943-024-01947-7

**Published:** 2024-02-17

**Authors:** Zongyao Huang, Yao Fu, Hong Yang, Yehan Zhou, Min Shi, Qingyun Li, Weiping Liu, Junheng Liang, Liuqing Zhu, Sheng Qin, Huangming Hong, Yang Liu

**Affiliations:** 1 Department of Pathology, Sichuan Clinical Research Center for Cancer, Sichuan Cancer Hospital & Institute, Sichuan Cancer Center, Affiliated Cancer Hospital of University of Electronic Science and Technology of China, Chengdu, China; 2 Genecast Biotechnology Co., Ltd, Wuxi, 214104 China; 3 Department of Pathology, West China Hospital, Sichuan University, Chengdu, 610041 Sichuan China; 4 Nanjing Geneseeq Technology Inc., Nanjing, 210032 Jiangsu China; 5 Department of Medical Oncology, Sichuan Clinical Research Center for Cancer, Sichuan Cancer Hospital & Institute, Sichuan Cancer Center, Affiliated Cancer Hospital of University of Electronic Science and Technology of China, Chengdu, China

**Keywords:** T-cell lymphoma, Liquid biopsy, cfDNA/ctDNA, CTCs, EBV DNA, Antibody, Cytokines

## Abstract

T-cell lymphoma is a highly invasive tumor with significant heterogeneity. Invasive tissue biopsy is the gold standard for acquiring molecular data and categorizing lymphoma patients into genetic subtypes. However, surgical intervention is unfeasible for patients who are critically ill, have unresectable tumors, or demonstrate low compliance, making tissue biopsies inaccessible to these patients. A critical need for a minimally invasive approach in T-cell lymphoma is evident, particularly in the areas of early diagnosis, prognostic monitoring, treatment response, and drug resistance. Therefore, the clinical application of liquid biopsy techniques has gained significant attention in T-cell lymphoma. Moreover, liquid biopsy requires fewer samples, exhibits good reproducibility, and enables real-time monitoring at molecular levels, thereby facilitating personalized health care. In this review, we provide a comprehensive overview of the current liquid biopsy biomarkers used for T-cell lymphoma, focusing on circulating cell-free DNA (cfDNA), circulating tumor DNA (ctDNA), circulating tumor cells (CTCs), Epstein-Barr virus (EBV) DNA, antibodies, and cytokines. Additionally, we discuss their clinical application, detection methodologies, ongoing clinical trials, and the challenges faced in the field of liquid biopsy.

## Introduction

T-cell lymphoma is a neoplasm characterized by highly invasiveness and notable heterogeneity. The most common subtypes of T-cell lymphoma include extranodal NK/T-cell lymphoma (ENKTL), nodal T-follicular helper cell lymphoma, angioimmunoblastic-type (nTFHL-AI), peripheral T-cell lymphoma, not otherwise specified (PTCL, NOS), and ALK-positive/negative anaplastic large cell lymphoma (ALCL), together accounting for 80% of T-cell lymphomas [1]. The emergence of precision medicine over the past decade has significantly enhanced our understanding of the biology, molecular subtypes, and genetic landscape of T-cell lymphoma [2, 3]. Furthermore, recent progress in basic and translational research has improved treatment options for lymphoma, including FDA-approved crizotinib for ALK-positive ALCL and Brentuximab Vedotin for CD30-positive T-cell lymphoma [4, 5]. Therefore, identifying patient subgroups at high risk of treatment failure, predicting early clinical outcomes during diagnosis or treatment, and optimizing patient selection for targeted therapy are now vital to modern translational lymphoma research and patient management.

Invasive tissue biopsy is the gold standard for histological diagnosis, facilitating the stratification of lymphoma patients into genetic subtypes and significantly contributing to treatment planning [6, 7]. However, tissue biopsy may not be suitable in several situations. For example, the risk associated with surgical intervention may lead to patient reluctance towards tissue biopsy. Also, patients with severe pre-existing disease or inaccessible tumors are restricted from undergoing surgical intervention, limiting their options for tissue biopsy. Moreover, tissue biopsy has inherent limitations. Given that the tissue biopsy is typically performed on a single tumor site and cannot be repeatedly sampled from the same patient throughout the course of the disease, it provides a limited perspective on the spatiotemporal heterogeneity in tumors [8, 9]. Additionally, tissue biopsies are not sensitive enough for detecting low levels of minimal residual disease (MRD) using imaging methods [10]. Therefore, the adoption of accurate, real-time technologies for the identification, measurement, and characterization of T cell lymphoma is essential to overcome these constraints and effectively implement the precision medicine approach in lymphoma treatment.

Liquid biopsy has emerged as an innovative and valuable approach in the field of non-invasive or minimally invasive diagnostics [11, 12]. This technique involves the extraction of biological information pertaining to malignancies from body fluids. The technology has exhibited noteworthy clinical applications and potential in various areas such as early diagnosis, prognosis assessment, treatment monitoring, resistance evaluation, MRD determination, and guiding treatment selection. Its advantages include non-invasiveness, the ability for multiple sampling, overcoming tumor heterogeneity, dynamic real-time monitoring, capturing additional mutation information compared to tissue-based genetic testing, and facilitating early recurrence detection. The predominant bodily fluid used for liquid biopsy is blood, but it can also include urine, cerebrospinal fluid, pleural or peritoneal effusion, saliva, feces, and semen. Liquid biopsy can be used to analyze a range of biomarkers present in bodily fluids, including circulating cell-free DNA (cfDNA), circulating tumor DNA (ctDNA), circulating tumor RNA, circulating tumor cells (CTCs), and viruses such as Epstein-Barr virus (EBV) [13–18]. MRD refers to a condition that the small number of cancer cells remain in the body during or after treatment. These residual cancer cells are typically undetectable by imaging or other standard diagnostic methods. Therefore, MRD assessment requires advanced techniques, such as flow cytometry (FCM) or next-generation sequencing (NGS), due to their higher sensitivity. MRD is a critical indicator of disease relapse in various cancers, including lymphoma [18]. Evidence indicates that cfDNA/ctDNA, CTCs, EBV DNA, and antibodies detected in liquid biopsy effectively define MRD status in patients with T-cell lymphomas [19–22].

**Fig. 1:**
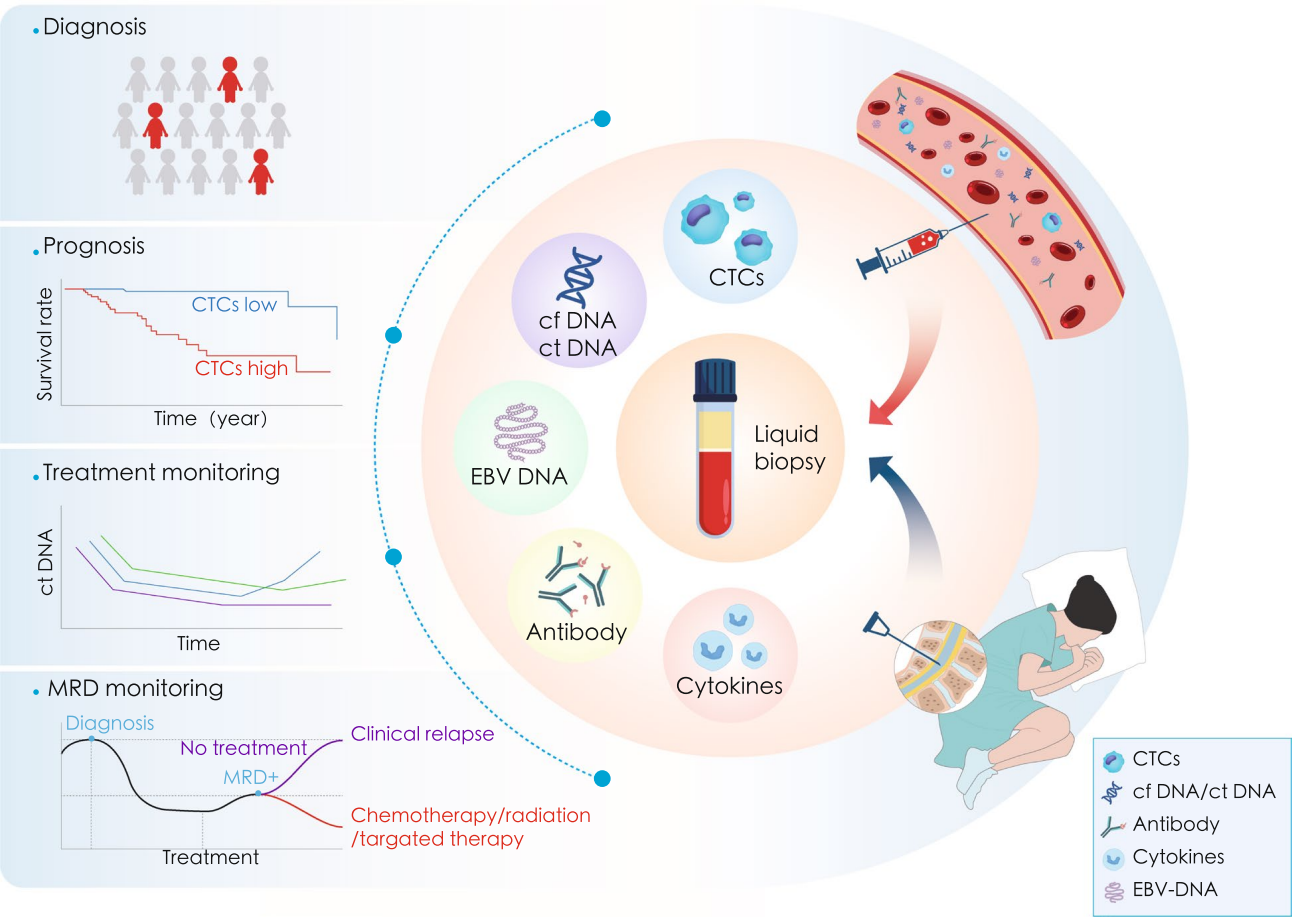
Application of liquid biopsy in T-cell lymphoma. Early diagnosis, prognostic prediction, treatment, and recurrence monitoring, as well as MRD assessment in T-cell lymphoma are all aided by the detection of various biomarkers, such as cfDNA/ctDNA (A), CTCs (B), EBV DNA (C), antibodies (D), and cytokines, using various techniques in liquid biopsy

**Fig. 2:**
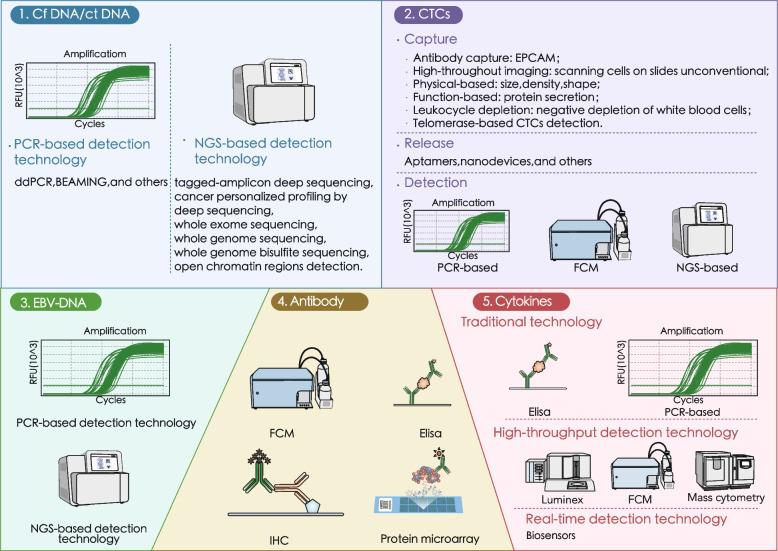
Overview of Liquid Biopsy Techniques in T-cell lymphoma. Different biomarker detection techniques of liquid biopsy for T-cell lymphoma include cfDNA/ctDNA, EBV DNA in CTCs, antibodies,cytokines

In this review, we provide a comprehensive overview of liquid biopsy biomarkers commonly used in T-cell lymphoma. We summarize the application of liquid biopsy in T-cell lymphoma, including its utility in early diagnosis, prognostic assessment, and treatment monitoring (Fig. 1). We also discuss the commonly used detection techniques in liquid biopsy and provide insights into the current clinical trials (Fig. 2). Furthermore, we address the challenges associated with the application of liquid biopsy in T-cell lymphoma.

## Liquid biopsy components

### cfDNA/ctDNA

ctDNA is a subset of cfDNA derived from tumor cells [19]. In healthy individuals, the plasma concentration of cfDNA ranges from 0 to 100 nanograms per milliliter, with an average of approximately 30 nanograms per milliliter. This concentration can be as high as 1000 nanograms per milliliter, with an average of 180 nanograms per milliliter of ctDNA extracted from blood or other bodily fluids in cancer patients [20]. Most cfDNA fragments are approximately 160–200 base pairs long, while ctDNA fragments are approximately 90–150 base pairs long [21]. Both are double-stranded fragments with a short half-life of approximately two hours [22, 23]. Subsequent studies have revealed that changes in tumor mutation burden, tissue damage, inflammation, and other factors affect the quantity and characteristics of ctDNA [24]. These characteristics enable ctDNA to monitor tumor occurrence and progression in real-time. Furthermore, ctDNA harbors tumor-specific genetic and epigenetic abnormalities, such as point mutations, structural variations, copy number variations, microsatellite alterations, open chromatine regions (OCRs), and methylation changes. These variations differ significantly among individuals [25]. The analysis of cfDNA and ctDNA is also utilized in the identification and detection of T-cell lymphoma. Table 1 presents a comprehensive compilation of the clinical applications of cfDNA and ctDNA as liquid biopsy biomarkers, specifically in T-cell lymphoma.

**Table 1:** Clinical application of cfDNA/ctDNA in T cell lymphoma

Lymphoma subtypes	Author	PMID/doi	Biomarker	Liquid Biopsy	Method	No. of patients	Results	Clinical applications
nTFHL-AI, PTCL, NOS	Sakata-Yanagimoto M et al. [26]	28634614	ctDNA	PB	NGS, 4 genes targeted sequencing	14	*TET2, RHOA G17V, DNMT3A,* and *IDH2* mutation of ctDNAs is a good candidate for diagnosis	diagnostic
nTFHL-AI	Hayashida, Masahiko et al. [27]	32476550	cfDNA	PB	AS-PCR	20	combination of multicolor FCM and *RHOA*^*G17V*^ and *IDH2*^*R172*^ hot-spot mutations in plasma cfDNA diagnosis of nTFHL-AI	diagnostic
nTFHL-AI, PTCL, NOS, ALCL	Ottolini, Barbara et al. [28]	32484856	cfDNA	PB	WES, NGS 81(CAPP-seq) and 12 genes targeted sequencing, ddPCR, Sanger sequencing	25	sequencing of *RHOA* from cfDNA has revealed new mutations and haplotypes *RHOA* c.73A > G (p.Phe25Leu) and c.48A > T (p.Cys16*)	diagnostic
ENKTL	Li, Qiong et al. [29]	32695399	ctDNA	PB	NGS, 41 genes targeted sequencing	65	mutated *KMT2D* and *ATM* predicted poor OS, *P* = 0.001, *P* = 0.001	prognosis
nTFHL-AI, ALCL, PTCL, NOS	Herrera, Alex F et al. [30]	27711974	ctDNA	PB	NGS, IGH VDJ, IGH DJ, IGK, TCR-β, TCR-δ, TCR-γ	68	84% patients had detectable ctDNA prior to progression at a median of 3.7 months. Patients with detectable ctDNA 3 months, 2-year PFS 58% vs. 84% *P* = 0.033)	longitudinal monitoring, MRD
PTCL, NOS, ALCL, nTFHL-AI, SS, SPTCL	Zhang, Wei et al. [31]	33734593	ctDNA	PB	mPCR, NGS, CDR3 of TCRβ chains	23	high level of ctDNA predicted treatment failure (*P* = 0.0003), ctDNA changed in concordance with clinical outcome and was more sensitive than PET/CT	longitudinal monitoring
PTCL, NOS, ALCL, nTFHL-AI	Miljkovic, Milos D et al. [32]	34432874	ctDNA(TCRβ, TCRγ)	PB	NGS, CDR3 of the TCRβ/TCRγ genes	45	patients with detectable ctDNA after therapy showed a trend toward worse survival. EFS, *P* = 0.083	longitudinal monitoring, MRD
T-LBL	Chen, Feili et al. [33]	32431543	ctDNA	PB	NGS, 102 genes targeted sequencing	43	92.6% agreement between ctDNA response and tumor volume measurements at post treatment when compared with baseline	longitudinal monitoring
ENKTL	Qi, Fei et al. [34]	34047776	cfDNA	PB	NGS, 112 genes targeted sequencing	24	cfDNA clearance vs no clearance, complete remission rates (80.0% vs 0%; *P* = 0.004), 1-year PFS, 79.0% vs 20.0%; *P* = 0.002). low cfDNA concentration before treatment (1-year PFS, 90.0% vs 36.4%; *P* = 0.012)	longitudinal monitoring, prognosis
PTCL, NOS, ALCL, nTFHL-AI	Kim, S.J. et al. [35]	35240014	ctDNA	PB	NGS, 66 genes targeted sequencing	94	≥ 1.5-log decrease in GE showed better PFS and OS, *P* = 0.027, *P* = 0.003	longitudinal monitoring

*cfDNA* circulating cell-free DNA, *ctDNA* circulating tumor DNA, *MRD* Minimal residual disease, *ALCL* Anaplastic large cell lymphoma.ENKTL, extranodal NK/T-cell lymphoma, *nTFHL-AI* Nodal T-follicular helper cell lymphoma, angioimmunoblastic-type, *PTCL, NOS* peripheral T-cell lymphoma, not otherwise specified, *T-LBL* T-cell lymphoblastic lymphoma, *SPTCL* subcutaneous panniculitis T cell lymphoma, *SS* Sézary syndrome, *NGS* Next-generation sequencing, *GE* genome equivalent, *WES* Whole-exome sequencing, *HTS* high-throughput sequencing, *AS-PCR* Allele-specific polymerase chain reaction, *RQ-PCR* Real-time quantitative PCR, *ddPCR* digital polymerase chain reaction, *CS-PCR* clonal specific PCR, *TCR* T-cell receptor, *PB* peripheral blood, *MDD* Minimal disseminated disease, *OS* overall survival, *DFS* disease-free survival, *PFS* progression-free survival

#### Application of cfDNA/ctDNA in liquid biopsy of t-cell lymphoma

Since ctDNA identifies tumor-specific gene alterations, it offers a non-invasive way for tumor diagnosis and assisting in precision treatment. Evidence has shown that epigenetic changes in *TET2, DNMT3A*, and *IDH2,* as well as the presence of *RHOA G17V* mutation, have diagnostic value for nTFHL-AI [36–38]. The *G17V RHOA* mutation in ctDNA from nTFHL-AI was further demonstrated to be 100% sensitivity and specificity in predicting the mutation status of tumor DNA [39]. These findings highlight the importance of ctDNA in nTFHL-AI for non-invasive diagnostic purposes. Additionally, targeted ctDNA detection also reveals tumor heterogeneity. Additionally, new mutations in exon 2 of *RHOA* in ctDNA, such as c.73A > G (p.Phe25Leu) and c.48A > T (p.Cys16*), were discovered in peripheral T-cell lymphoma [40]. These studies indicate that disease-specific gene mutations are sensitive indicators of ctDNA, which is crucial for precise treatment. Therefore, ctDNA testing in liquid biopsy offers a promising diagnostic method for T-cell lymphoma, particularly when invasive tissue biopsies are not feasible.

cfDNA/ctDNA is a valuable biomarker for predicting the prognosis and recurrence of T-cell lymphoma. In a prospective study [41], targeted NGS was performed on paired tumor tissue and a series of longitudinal plasma cfDNA samples from ENKTL patients. The study demonstrated the concordance between the genotyping results of paired baseline tumor tissue and cfDNA. It also showed the prognostic value of cfDNA concentration, demonstrating that ENKTL patients had better survival outcomes when their cfDNA concentration was low (one-year PFS: 90.0% vs. 36.4%; *P* = 0.012). Furthermore, certain genetic mutations identified from ctDNA are significantly associated with poor prognosis, thereby improving the predictive ability of tumor prognosis [42].

An anthracycline-based chemotherapy regimen is the standard first-line treatment for most T-cell lymphomas, yet it only benefits a subset of patients. The 5-year survival rates for PTCL, NOS, nTFHL-AIs, and ALK-positive ALCLs have been reported to be 32%–43%, 32%–36%, and approximately 70%, respectively. A primary cause of first-line treatment failure is treatment resistance. Therefore, the adoption of liquid biopsy during treatment can aid in the identification of responsive patients, allowing physicians to adjust treatment plans promptly and effectively [4, 43]. cfDNA/ctDNA is beneficial for dynamically monitoring disease progression when obtaining tissue biopsy samples is not feasible. In a longitudinal study of ctDNA in 45 patients with diverse subtypes of PTCL, Seok Jin Kim et al. found that post-treatment ctDNA mutation burden was strongly correlated with disease recurrence or progression [44]. Additionally, a decrease in genomic equivalents (GE) of over 1.5log from baseline to the end of treatment was significantly associated with improved survival outcomes, compared to a decrease less than 1.5log [45]. These findings demonstrate the important clinical utility of detecting and monitoring cfDNA/ctDNA for dynamic monitoring and evaluation of treatment efficacy in T-cell lymphoma.

Previous studies have demonstrated the role of cfDNA/ctDNA in MRD assessment in lymphoma after treatment [45, 46]. Several studies have shown a trend toward decreased survival rates in patients with detectable ctDNA after treatment in T-cell lymphomas. Herrera et al. [47] evaluated the association between the presence of MRD and relapse/progression in lymphoma patients undergoing allogeneic hematopoietic stem cell transplantation (HSCT). In their study, 16 out of 19 patients (84%) had detectable ctDNA within 3.7 months prior to their relapse/progression. However, not all patients with post-HSCT ctDNA detection had relapse, suggesting a possible distinction between residual disease and actual relapse/progression. The progression-free survival (PFS) rates for patients with detectable ctDNA at three months post-HSCT were lower compared to ctDNA-negative patients (two-year PFS: 58% vs. 84%). Multivariant analysis showed that detectable ctDNA was associated with increased risk of progression/death (hazard ratio 3.9) and increased risk of relapse/progression (hazard ratio 10.8). In another study [48], the utility of NGS-based ctDNA monitoring for MRD assessment was investigated in PTCL patients undergoing frontline therapy. They found that 13 out of 24 patients (54%) cleared ctDNA after treatment, while 11 patients (46%) had detectable ctDNA at the end of treatment. Patients with detectable ctDNA after treatment had a median survival time of one year, whereas patients without detectable ctDNA after treatment did not reach the median survival time [48]. Another longitudinal study of ENKTL showed that patients with persistently undetectable ctDNA after treatment had a significantly higher complete remission rate than patients with persistently detectable ctDNA after treatment (80% vs 0%; *P* = 0.004) [41]. Therefore, the detection of cfDNA/ctDNA plays a crucial role in defining the MRD status of T-cell lymphoma, providing significant insights into prognosis and treatment efficacy.

#### Methods for collection, quantification, and detection of cfDNA/ctDNA

The practicality of liquid biopsy techniques relies heavily on achieving sufficient sensitivity and specificity to detect trace amounts of cfDNA/ctDNA in body fluids. The technical performance of liquid biopsy methods has significantly improved because of recent advancements in molecular biology, high-throughput analysis, and bioinformatics that have surmounted important barriers, including pre-analytical problems [49, 50]. Prior to conducting liquid biopsy analysis in lymphoma, optimal sample collection and processing of cfDNA/ctDNA are essential considerations. For instance, liquid biopsy typically requires only a small volume of blood, usually between 6 to 10 ml [20]. However, the actual amount of plasma needed depends on various factors, including the patient's condition, the detection techniques used in the laboratory, and the sensitivity of the assay [20, 51]. Additionally, given that the half-life of ctDNA is approximately two hours [52], it is crucial to promptly separate plasma from blood collection in EDTA tubes to prevent contamination with cfDNA released by peripheral blood mononuclear cells [53]. Several commercial cfDNA preservative tubes are used in practice to stabilize nucleated blood cells. Examples include BD Vacutainer K2E EDTA spray tubes, Streck Cell-Free DNA BCT tubes, PAXgene Blood cfDNA tubes, Roche Cell-Free DNA Collection tubes, and Biomatrica LBgard blood tubes, among others. Studies have found [54–56] that blood collected in cfDNA preservation tubes can be stored for up to 3–7 days at room temperature without affecting DNA yield or mutation background levels. It is generally believed that cfDNA is stable for up to 14 days at 6 °C to 37 °C, while CTCs are stable for up to seven days at room temperature, allowing convenient sample collection, transport, and storage.

Sensitive and specific detection methods are required due to the low concentration and short half-life of cfDNA/ctDNA in the body [57]. The primary techniques used are PCR and NGS. PCR is a widely used method with high sensitivity and specificity for the detection of single molecular abnormalities. PCR-based techniques include droplet digital PCR (ddPCR) and beads, emulsion, amplification, and magnetics (BEAMing) [58–60]. ddPCR can detect genomic material as low as 0.01–1.0% and can be used to identify rare mutations and calculate copy number variations. However, for simultaneous detection of multiple targets, ddPCR typically employs multiple fluorescence detection channels. Each channel corresponds to a specific target sequence; binding of amplified DNA to a corresponding fluorescent probe generates a signal in that channel. By analyzing signals from different fluorescent channels, different targets can be distinguished and quantified. At present, most commercial digital PCR platforms are equipped with 2–6 fluorescent channels, so only two to six targets can be detected simultaneously in one reaction, thus limiting their use to assess hotspot mutations only [61]. BEAMing, combining PCR with FCM, is a relatively sensitive and cost-effective detection method for known mutations. It can detect changes as low as 0.01% and shows good concordance with tissue testing [62].

NGS-based detection techniques can be used for the detection of various molecular abnormalities, including targeted sequencing and untargeted sequencing. Targeted sequencing methods include tagged-amplicon deep sequencing (TAm-Seq) and cancer personalized profiling by deep sequencing (CAPP-Seq) [63, 64]. Untargeted NGS methods include whole-exome sequencing (WES), whole-genome sequencing (WGS), and whole-genome bisulfite sequencing (WGBS-Seq) [65, 66]. WGS evaluates the entire tumor genome to identify characteristic and pathogenic alterations, as well as variants of uncertain significance. While it provides a comprehensive assessment of all tumor mutations, it is constrained by factors such as quality assurance, ethical considerations, time, and cost.WES detects mutations only in the exon regions of the human genome, allowing for the identification of potential oncogenes and tumor suppressor genes. Consequently, WES has a cost advantage over WGS. However, its sensitivity may be lower compared to WGS [65]. Additionally, interpretation of the sequencing results from these two methods may be challenging outside of specialized centers [67]. WGBS-Seq is considered the gold standard for DNA methylation analysis. It provides highly accurate measurements of individual cytosines. While capable of detecting partially methylated regions in cancer cells, its sensitivity is limited due to the potential for DNA degradation [68]. Additionally, specific detection of DNA hypermethylation in promoter regions can be evaluated using methylation-specific PCR [69].

In recent years, some new methods have gradually known by public. The transcription and replication of chromatin in eukaryotes require a combination of specific cis-regulatory elements and trans-acting factors for regulation. OCRs are the regions of the human genome accessible to DNA regulatory elements [70, 71]. The analysis of cfDNA fragment characteristics has advanced our understanding, enabling us to infer gene expression and predict transcription factor binding sites (TFBS) [72]. Generally, genes that are actively expressed are associated with OCRs. Although no research has reported the association between OCRs and T-cell lymphoma, cfDNA sequencing has the potential to detect OCRs. Wang, Jiayin et al. [72, 73] proposed a novel bioinformatics pipeline, OCRDetector, which demonstrated a good performance in detecting OCRs from whole genome cfDNA sequencing data. It is achieved by calculating the window protection score (WPS) waveform and the cfDNA sequencing coverage. The OCRDetector method effectively utilizes the characteristics of cfDNA fragments to address the challenge of genome-wide waveform pattern recognition. It improved the measurement of chromatin accessibility of the whole genome, providing a foundational bioinformatics pipeline for future research.

In summary, sensitive and specific detection methods, including PCR- and NGS-based techniques, are required for cfDNA/ctDNA detection in liquid biopsies. These methods play crucial roles in diagnosis, prognosis, treatment response evaluation, and MRD assessment in lymphoma.

### CTCs

CTCs are tumor cells that are shed into the bloodstream from primary or metastatic tumor sites [74]. They may be released as clusters from the primary source or found as individual cells. Their presence has been associated with overall survival (OS) and prognosis [75]. CTCs provide early insights into the primary tumor, such as genomic alterations [76] and gene expression profiles characterizing tumor cells [77]. CTCs are highly heterogeneous, allowing them to easily evade immune surveillance and treatment, ultimately leading to distant metastasis or tumor cell recurrence [74]. Due to the short half-life of CTCs in the blood(1–2.4 h), the concentration is low [78]. Therefore, the detection of CTCS in T-cell lymphomas is a great challenge. However, new technologies can still identify the types and quantities of CTCs, thereby aiding in early diagnosis, prognostic stratification, dynamic assessment, and defining MRD status of the tumor. The clinical applications of CTCs as liquid biopsy biomarkers in T-cell lymphoma are summarized in the table below (Table 2).

**Table 2:** Clinical application of CTCs in T cell lymphoma

Lymphoma subtypes	Author	PMID/doi	Biomarker	Liquid Biopsy	Method	No. of patients	Results	Clinical applications
CTCL, PTCL, NOS, T-LBL, nTFHL-AI	Assaf, C et al. [79]	10887129	CTCs	PB	PCR, TCR-beta	62	TCR-gamma PCR revealed an approximate 20% lower detection rate in the same set of samples than with the TCR-beta PCR method	diagnostic
PTCL, NOS, nTFHL-AI, ALCL, ENKTL	Pu Q et al. [80]	36466894	CTCs	PB	FC, 15 markers	81	NTFHL-AI: PD-1 + % > 38.01, CD10 + % > 7.46 ENKTL-N: CD56 ALCL: CD30 and HLA-DR P MRD sensitivity, 85.71% prognostic: NTFHL-AI, CD7, CD38, Ki-67 PTCL, NOS, CD3, CD8 ENKTL, CD56 and HLA-DR	diagnostic, prognosis,
ALK-positive ALCL	Damm-Welk, Christine et al. [81]	17392503	CTCs	PB	RT-PCR, RQ-PCR, *NPM::ALK* expression	80	*NPM::ALK*: 47.5% CI-Rs: 38 pts PCR-positive, 50% ± 10% vs 42 pts PCR-negative, 15% ± 7% (*P* < 0.001) CI-R: 16 pts *NPM*::*ALK* > 10 NCNs, 71% ± 14% vs 59pts ≤ 10 NCNs, 18% ± 6% (*P* < 0.001)	prognosis
ALK-positive ALCL	Damm-Welk, Christine et al. [82]	31649129	CTCs	PB	dPCR, RQ-PCR *NPM*::*ALK*	91	Applying a cut-off of 30 copies *NPM::ALK*/10^4^ copies *ABL1* for quantification by dPCR, almost identical groups of patients were separated as those separated by RQ-PCR	prognosis
ALCL	Mussolin, Lara et al. [83]	32987765	CTCs	PB	qualitative PCR, *NPM::ALK*	420	high-risk group (bpHR): MDD-positive & SC/LH pattern; 10-year PFS 40% low-risk group (bpLR): MDD-negative & without SC/LH pattern; 10-year PFS 86% intermediate-risk group (bpIR): remaining patients. 10-year PFS 75% (*P* < 0.0001)	prognosis
T-LBL	Coustan-Smith E et al. [84]	19546402	CTCs	PB	FC	99	Two-year EFS: patients ≥ 1% T-LL cells in bone marrow vs < 1% vs ≥ 5%, 68.1% ± 11.1% (SE) vs 90.7% ± 4.4%(*P* = .031) vs 51.9% ± 18.0% (*P* = .009)	diagnostic Prognosis longitudinal monitoring
PTCL, NOS, ALK-negative ALCL, nTFHL-AI	Gauthier, J et al. [85]	27775693	CTCs	PB	mpFC, 10-colour FC, TCR V-beta,	29	7/29 patients were MDD + .pre-ASCT MDD + OS(19% vs 89%, P < 0.001), CIR, 85% vs 36%, P < 0.001)	prognosis
ALK-positive ALCL	Kalinova M et al. [86]	17320171	CTCs	PB	RQ-RT-PCR, *NPM::ALK*	10	RQ-RT-PCR revealed *NPM::ALK* positivity 100% from relapses and/or closely before a relapse in at least one of the iliac BM trephines	MRD
ALK-positive ALCL	Damm-Welk, Christine et al. [87]	24297868	CTCs	PB	RT-PCR, *NPM::ALK*	180	MDD + : 103/180. MRD + :52(before the second therapy course in 52 MDD + pts) CIR: 26 MDD + /MRD + pts (81% ± 8%) vs 26 pts MDD + /MRD- (31% ± 9%) vs 77 pts MDD- (15% ± 5%) (*P* < 0.001) Five-year survival: MDD- vs MDD + /MRD- vs MDD + /MRD + pts: 91% ± 3% vs 92% ± 5% vs 65% ± 9% (*P* < 0.001)	MRD
ALK-positive ALCL	Rigaud, Charlotte et al. [88]	33687135	CTCs	PB	multiplex RT-PCR, *NPM1*::*ALK*	138	diagnosis: 87/138 pts MDD + Early MRD (before the second course (BM1)) + : 18/33, MRD-:15/33 Three-year PFS:81.1% in MDD − , 69.6% in MDD + /MRD − , 15.2% in MDD + /MRD + pts	MRD
ALK-positive ALCL	Pokorna P et al. [89]	35700412	CTCs	PB	RT-PCR, *NPM1*::*ALK*	1	intermittent dosing of lorlatinib improved treatment response and prolongation of the child’s EFS. MRD dynamics supported the selected administration schedule	MRD
CTCL	Wolfe, J T et al. [90]	7602365	CTCs	PB	PCR, T-cell receptor-beta (TCR-beta) rearrangements	1	a complete clinical response CTCL patient detected MRD when clinical relapsed identified a common clonal origin for CTCL and LCT	MRD
ATLL, CTCL, T-LGLL, PTCL, NOS	Tembhare P et al. [91]	22261447	CTCs	CSF, FNA	FCM, TCR-V(β)-R	8	6 CSF FCM + , 1 was positive by cytomorphologic evaluation, 3 were negative, 2 were suspicious or atypical All FNA specimens were FCM + . 4/8 were positive by cytomorphologic evaluation and four were suspicious or atypical	MRD
ATLL	Shao, Haipeng et al. [92]	20231613	CTCs	PB	FC, 25 marker PCR: TRG Gene Rearrangements and HTLV-1	17	FC: 0.29% ATLL cells/WBC (4.9 cells/microL) PCR: TRG gene rearrangement, 74% demonstrated. 14% suspicious; HTLV-1,96% MRD: FC & PCR: 30/50, 60%	MRD
ATLL, T-LGLL, PTCL, NOS, T-PLL	Tembhare, Prashant et al.[93]	21571962	CTCs	PB	FC, TCR-Vβ	41	TCR-V(β)-R analysis is a sensitive method for detection of MRD (14/14,100%)	diagnostic MRD
T-ALL	Wu, David et al. [94]	22593176	CTCs	PB	mpFC, 18 marker HTS, TCRG and TCRB CDR3	43	diagnosis: TCRB-HTS: 31/43, TCRG-HTS: 27/43 MRD: TCRB-HTS & CRG-HTS: 25/35 mpFC: 13/35	MRD
MF SS	Weng, Wen-Kai et al. [95]	24307695	CTCs	PB	mpFC, 7-marker, TCRβ HTS, TCRβ	10	malignant clones were detected by TCRB-HTS in all ten cases, four of these patients achieved molecular clearance in skin after transplant	MRD

*CTCs* circulating tumor cells, *MRD* Minimal residual disease, *ATLL* adult T-cell leukemia/lymphoma, *T-LGLL* T-large granular lymphocytic leukaemia. T-PLL, T-prolymphocytic leukemia, *ALCL* Anaplastic large cell lymphoma, *nTFHL-AI* Nodal T-follicular helper cell lymphoma, angioimmunoblastic-type, *PTCL, NOS* peripheral T-cell lymphoma, not otherwise specified, *T-LBL* T-cell lymphoblastic lymphoma, *T-ALL* T-cell lymphoblastic leukemia, *CTCL* cutaneous T-cell lymphoma, *ENKTL* extranodal NK/T-cell lymphoma, *NGS* Next-generation sequencing, *GE* genome equivalent, *WES* Whole-exome sequencing, *HTS* high-throughput sequencing, *AS-PCR* Allele-specific polymerase chain reaction, *RQ-PCR* Real-time quantitative PCR, *dPCR* digital polymerase chain reaction, *CS-PCR* clonal specific PCR, *TCR* T-cell receptor, *SC/LH* small cell/lymphohistiocytic, *LCT* large-cell transformation, *CI-Rs* cumulative incidence of relapses, *PB* peripheral blood, *MF* Mycosis fungoides, *SS* Sézary syndrome, *CSF* cerebrospinal fluid, *MDD* Minimal disseminated disease, *FNA* fine-needle aspirate, *MFC* multicolor flow cytometric, *mpFC* Multiparameter flow cytometry, *OS* overall survival, *DFS* disease-free survival. *PFS* progression-free survival, *BM* bone marrow

#### Application of CTCs in liquid biopsy of T-cell lymphoma

In T-cell lymphoma, differentiating between malignant lymphoma and reactive lymphoproliferative lesions can be challenging, or even impossible, using the conventional histological method alone. CTCs are widely recognized as important determinants of cancer metastasis and recurrence [96]. Despite the emergence of several monoclonal antibodies that improve the identification of T-cell subsets, distinguishing between non-neoplastic and neoplastic T cells remains a challenge. T-cell receptor (TCR) gene rearrangements are often regarded as valuable tools for identifying malignant T-cell proliferation. In normal and reactive proliferative T cells, these genes are rearranged in different ways (i.e., polyclonal), while in T-cell lymphoma, the tumor cells contain monoclonal TCR gene rearrangements. A controlled study used flow cytometric immunophenotyping (TCR-Vβ-R) to detect T-cell clonality in liquid samples. This method successfully identified abnormal T-cell populations and their immunophenotypes, aiding in disease diagnosis [73]. Similarly, Qiyao Pu’s research team utilized FCM-based detection of peripheral blood immunophenotypic markers in PTCL. This approach identified and diagnosed various subtypes, demonstrating significant clinical and differential diagnostic value [97]. Therefore, CTCs can serve as a promising biomarker for both diagnosis and differential diagnosis in T-cell lymphoma.

CTCs are one of the most important prognostic and treatment monitoring biomarkers in solid tumors and lymphomas [98, 99]. There is a significant correlation between CTC counts and patient survival. A study involving 99 children with T-cell lymphoblastic lymphoma (T-LBL) found that a worse outcome was linked to higher levels of CTCs detected in peripheral blood or bone marrow before treatment [100]. Additionally, they monitored residual cancer cells in the blood during remission induction therapy. This monitoring identified patients with slower disease clearance and determined the presence of MRD through CTC measurement. They also found that the presence of MRD during treatment was associated with a worse prognosis. In another study [101] involving 180 patients with ALK-positive ALCL who underwent second-line therapy, patients defined as MRD-positive based on CTCs had significantly lower 5-year survival rates and event-free survival compared to MRD-negative patients, and had a higher risk of relapse. Therefore, early assessment of MRD in ALK-positive ALCL allows for the identification of patients with a high risk of relapse and lower survival rates.

In summary, CTCs hold great promise in the fields of non-invasive diagnosis, prognosis assessment, real-time monitoring of treatment efficacy, and defining MRD status. Their ability to provide early tumor information and dynamic changes makes them a valuable tool for liquid biopsy, overcoming the limitations of tissue biopsies. Monitoring the quantity and characteristics of CTCs enables evaluation of patient prognosis and early detection of poor treatment response or recurrence. Additionally, CTCs can be utilized to define MRD status, aiding in the accurate assessment of treatment response and guiding subsequent treatment strategies.

#### Capture, release, and detection of CTCs

Isolation of normal cells and extraction of tumor cells from the abundant blood components in cancer patients are challenging, as most patients with metastatic cancer have fewer than 10 CTCs per ml of blood. Consequently, techniques to capture intact CTCs from the circulation present considerable obstacles [78]. The heterogeneity of CTCs further complicates their isolation. Therefore, establishing standardized detection methods for CTCs, along with research into innovative technologies, will improve the sensitivity and accuracy of early malignant tumor diagnosis [102].

Currently, several notable capture technologies for CTCs exist. These include affinity-based methods represented by methods, such as EpCAM selection on solid tumors or lymphoma cells [98, 103]; high-throughput imaging methods such as cell scanning on slides [104]; approaches based on physical properties based on tumor size, shape, density, etc. [105, 106]; methods focusing on functional characteristics, such as protein secretion, migration properties [107, 108]; leukocyte depletion by negative selection of white blood cells [109, 110]. New studies have also utilized the activation characteristics of telomerase in cancer cells and developed telomerase-based CTCs detection to detect CTCs [111–113]. After cell capture, CTCs must be released in an effective manner that allows the cells to remain intact and viable for culturing and analysis. Aptamers, nanodevices, and unconventional methods involving optical, electrical, and chemical processes have been used for CTC isolation [114–117].

From a clinical perspective, genetic profiling of CTCs may be one of the most direct applications. The use of allele-specific PCR for detecting CTC-enriched cell populations has proven effective in T-cell lymphoma, with a high concordance observed between tumor biopsy and CTC-derived genotyping [97, 118]. Allele-specific PCR or targeted NGS analysis can also be used to record the emergence of therapy-related resistance mutations. However, the application of WES is complicated by the very low levels of tumor-specific template and contamination from leukocyte-derived sequences [119]. Advances in NGS strategies and computational analysis may effectively address this challenge. FCM has been increasingly attractive as a technique for CTCs detection in T-cell lymphoma [120–122], primarily for identifying immunophenotypically abnormal cell populations. Additionally, studies have shown that CTC quantification using FCM outperforms bone marrow tissue-based CTC quantification [123]. Overall, while each detection technology has its advantages and disadvantages, integrating various techniques in the selection process can enhance the detection capability of CTCs [124].

### EBV DNA

EBV was first discovered in 1964 by Epstein et al. in the Burkitt lymphoma biopsy cells [125]. Since then, extensive research on this virus has greatly contributed to the advancements in tumor biology, tumor immunology, molecular virology, clinical virology, and viral diagnostics. EBV DNA refers to the DNA fragments secreted by the EBV in the body and is commonly used in liquid biopsies to detect EBV-related diseases, such as malignant lymphoma and nasopharyngeal carcinoma [126]. EBV DNA can be actively released or squeezed out from viable cells, or it can be passively shed from cells during cell apoptosis or necrosis. The direct and quantitative detection of minute residual viral DNA holds significant importance in disease diagnosis and monitoring. In nasopharyngeal carcinoma, circulating cell-free EBV DNA is considered an established tumor biomarker [127, 128]. Increasing evidence suggests the potential clinical utility of liquid biopsy-based EBV DNA as a biomarker in EBV-related Hodgkin lymphoma and EBV-related T-cell lymphoma [129–131].

In this review, we summarize the clinical applications of EBV DNA as a liquid biopsy biomarker in T-cell lymphoma. These applications include diagnosis, prognosis assessment, treatment monitoring, and defining MRD status (Table 3).

**Table 3:** Clinical application of EBV DNA in T cell lymphoma

Lymphoma subtypes	Author	PMID/doi	Biomarker	Liquid Biopsy	Method	No. of patients	Results	Clinical applications
ENKTL	Lei, Kenny I K et al. [132]	11801537	EBV DNA levels	PB	qRT-PCR	18	plasma EBV DNA was detected in 17/18 patients but in 0/35 control subjects (*P* < 0.0001) Clinically responding patients plasma EBV DNA levels fall whereas no responding patients rapid increase patients baseline plasma EBV DNA levels ≥ 600 copies/ml vs < 600 copies/ml, OS 21% vs 78%; *P* = 0.024	diagnosis, prognosis. longitudinal monitoring
ENKTL, nTFHL-AI	Au, Wing-Yan et al. [133]	15031209	EBV DNA levels	PB	Q-PCR	39	high-presentation EBV DNA (> 6.1 × 10^7^ copies/mL) was significantly associated with an inferior DFS. During treatment, patients with EBV DNA had significantly inferior OS	diagnosis, prognosis. longitudinal monitoring
ENKTL	Kwong YL et al. [134]	23842425	EBV DNA levels	PB	Q-PCR	56	negative EBV DNA after SMILE had the most significant impact (*P* < 0.001) on OS and persistently undetectable EBV DNA had the most significant impact (*P* = 0.002) on DFS	prognosis
ENKTL	Wang XX et al. [135]	30646796	EBV DNA levels	PB	qRT-PCR	99	3-year OS: pre-, interim-, post-treatment EBV-DNA, positive vs negative, 70.2% vs. 93.9% (*P* = 0.022), 53.8% vs. 99.1% (*P* < 0.001), 40.6% vs. 91.8% (*P* < 0.001)	prognosis. longitudinal monitoring
PTCL, NOS, ALCL, nTFHL-AI	Zhao D et al. [136]	34753127	EBV DNA levels	PB	qRT-PCR	128	pre-treatment EBV-DNA ≥ 500 copies/mL(3-year PFS: 12.2% vs. 48.2%, *p* = 0.001; 3-year OS: 42.5% vs. 66.1%, *P* = 0.003) EBV-DNA with normalization of after first-line chemotherapy ORR (81.3% vs. 22.2%, *P* = 0.014), PFS (12.0 m vs. 3.7 m, *P* = 0.011), OS (37.9 m vs. 7.8 m, *P* = 0.012)	prognosis. longitudinal monitoring
PTCL, NOS, nTFHL-AI, ENKTL	Suwiwat, Supaporn et al. [137]	17996493	EBV DNA levels	PB	RT-PCR	45	concentration of EBV DNA in the plasma was not a prognostic marker in PTCL and PTPD patients	prognosis
nTFHL-AI	Delfau-Larue, MH et al. [138]	22371178	EBV DNA levels	PB	RT-PCR	25	peripheral EBV load at diagnosis (≥ 100 copy/μg DNA) was associated with shorter PFS (*P* = 0.06)	prognosis
ENKTL	Wang ZY et al. [139]	22826562	EBV DNA levels	PB	RT-PCR	69	3-year OS and PFS rates: pretreatment EBV-DNA level of ≤ 500 copies/mL vs > 500 copies/mL, 97.1% and 79.0% vs 66.3% (*P* = .002) and 52.2% (*P* = .045)	prognosis
ENKTL	Wang L et al. [140]	26210287	EBV DNA levels	PB	qRT-PCR	68	Patients with negative pretreatment EBV-DNA had a higher CR rate (96.0% vs. 69.8%, *P* = 0.023) post-treatment EBV-DNA positivity and treatment response (non-CR) were prognostic factors for both worse PFS and OS (*P* < 0.05)	prognosis MRD

*EBV* Epstein-Barr virus, *EBV DNA* Epstein-Barr virus DNA, *ENKTL* extranodal NK/T-cell lymphoma, *PTCL, NOS* peripheral T-cell lymphoma, not otherwise specified, *nTFHL-AI* Nodal T-follicular helper cell lymphoma, angioimmunoblastic-type, *PTPD* peripheral T-cell proliferative diseases, *qRT-PCR* real-time quantitative PCR, *OS* overall survival, *DFS* disease-free survival, *PFS* progression-free survival

#### Application of EBV DNA in liquid biopsy of T-cell lymphoma

EBV DNA has the potential to serve as a diagnostic biomarker for EBV-related tumors. In a study conducted by Lei et al. [141], qRT-PCR was used to detect EBV DNA in the blood of 18 patients with NK/T-cell lymphoma. Seventeen out of the 18 patients tested positive for plasma EBV DNA (median: 659 copies/mL), while none of the 35 healthy controls showed detection. Another independent study included 39 lymphoma patients who were EBV-positive prior to treatment [142]. Following treatment, the levels of EBV DNA showed dynamic changes. However, among 34 cases of EBV-negative lymphoma, no EBV DNA was detected in the body fluids during diagnosis, chemotherapy, or at any disease stage. These findings suggest that EBV DNA has the potential to serve as a diagnostic biomarker in EBV-related lymphoma. However, before its implementation as a diagnostic marker, it's crucial to determine if the detection technique can differentiate between tumor- and non-tumor- derived EBV DNA. Also, it is important to accurately identify EBV DNA derived from PTCL and recognize viral DNA gene sequences with diagnostic relevance [21, 123, 126, 127].

Studies have suggested that EBV DNA levels in body fluids are significantly correlated with OS and disease-free survival. EBV DNA, reflecting tumor burden, can effectively predict prognosis and serve as a prognostic biomarker for EBV DNA-related lymphomas [141–143]. However, this conclusion remains controversial. For example, a study found that in individuals with EBV-related lymphomas and peripheral T-cell proliferative diseases, plasma EBV DNA concentration neither served as a predictive marker nor was associated with survival outcomes [144]. These conflicting observations have led ongoing clinical trials to directly incorporate plasma EBV DNA levels into their study designs, such as NCT04676789.

Plasma EBV DNA in circulation can be utilized for monitoring the effectiveness of tumor treatment and defining MRD status. This approach may also be applicable to the analysis of ctDNA in EBV-related lymphomas [145]. Circulating EBV DNA serves as a precise biomarker for tumor burden in ENKTL [146, 147]. A study evaluated the role of plasma EBV DNA in monitoring the treatment of ENKTL patients [148]. Patients receiving P-GEMOX treatment had their plasma EBV DNA levels quantitatively evaluated before, during, and after treatment. The study found that dynamic changes in plasma EBV-DNA levels were associated with clinical responses during treatment. Plasma EBV-DNA is also crucial for evaluating residual tumor burden and defining MRD status after treatment [149, 150]. In patients with EBV-DNA-positive ENKTL before treatment, a negative EBV-DNA status after treatment was correlated with better PFS and OS, whereas a positive EBV-DNA status after treatment was associated with a higher rate of relapse and progression [146]. These findings suggest that EBV DNA can serve as an indicator for MRD. Therefore, liquid biopsy of EBV DNA is particularly suitable for post-treatment evaluation in patients, due to its convenient, non-invasive, and real-time characteristics. This is especially important, as obtaining fresh post-treatment samples poses significant challenges, even for experienced pathologists.

#### Detection of EBV DNA

In T-cell lymphoma patients, PCR-based approaches are typically used to estimate viral load and measure plasma EBV DNA. Many of these PCR-based methods target specific regions of the EBV genome, such as *Pol-1* and *Lmp2* [151]. A meta-analysis evaluated the diagnostic role of EBV DNA detection and quantification in the serum of pediatric and young adult patients with infectious mononucleosis [152]. The sensitivity of plasma PCR-based EBV DNA detection was found to be 77%, with a specificity of 98%. The results demonstrate that PCR-based detection of EBV DNA has high specificity and good sensitivity. Therefore, it is currently the common approach and nursing standard for EBV DNA detection [143, 146, 153].

Although PCR-based EBV detection is widely accepted as the nursing standard in many institutions, it has certain limitations. The lack of unified EBV DNA testing has hindered its application in clinical practice. Variability between different PCR detection techniques remains a challenge [154], including differences in amplicon size determination, relative quantification estimation (describing the threshold for reporting positive results), and inconsistencies in standards used for PCR calibration. All these factors can affect critical assay features such as sensitivity, specificity, and detection limits. In contrast, NGS offers numerous advantages for detecting tumor-associated viral DNA. Firstly, sequencing-based methods reveal the sequences of EBV DNA, which can help better distinguish EBV derived from PTCL and identify viral DNA gene sequences with prognostic or predictive significance. Secondly, sequencing assesses DNA size, which offers additional approach to differentiate tumor derived EBV fragments from non-tumor EBV DNA. Thirdly, PCR-based detection relies on amplifying target amplicon regions within the viral genome, while sequencing-based methods allow for quantification of any EBV DNA fragment mapping to the viral genome. Fourthly, sequencing-based detection theoretically allows for a broader dynamic range; therefore, it improves sensitivity and specificity of target detection by more accurately determining absolute EBV DNA abundance and integrating information about the sequences, including content and fragment size [131, 153, 155]. However, NGS also has limitations, such as gene information mismatch, incomplete detection information, and false positives, restricting its widespread application [156].

In addition to DNA sequencing, whole-genome methylation analysis has also demonstrated disease-specific EBV epigenetic profiles between NPC, lymphoma, and infectious mononucleosis patients [157]. The integration of DNA sequencing and methylation profiles has improved the positive predictive value of plasma EBV DNA-based lymphoma diagnosis. Future research could focus on expanding this analysis to larger cohorts, incorporating other genomic methods, and developing novel and complementary approaches for plasma EBV DNA detection.

### Antibodies

Antibodies are recognized as significant biomarkers for various diseases. the human immune system produces specific antibodies to counteract external antigens in many human diseases, such as tumors [158], inflammation [159], and neurological and psychiatric disorders [160]. Therefore, quantified antibodies serve as reliable indicators of disease progression [161]. The abundance of antibodies in human blood, combined with the ease of blood processing, facilitates the collection and identification of these biomarkers [154]. Consequently, antibody quantification plays a vital role in monitoring potential treatment toxicity and assessing therapeutic response [162].

In lymphoma research, the utilization of antibody reactions primarily involves the detection of ALK antibody titers in peripheral blood and/or bone marrow samples of ALK-positive ALCL patients. This approach is researched for their diagnostic applications, prognostic value, and utility in MRD assessment. Table 4 summarizes studies focused on antibody detection in liquid biopsies.

**Table 4:** Clinical application of antibody in T cell lymphoma

Lymphoma subtypes	Author	PMID/doi	Biomarker	Liquid Biopsy	Method	No. of patients	Results	Clinical applications
ALCL	Mussolin, L et al. [163]	18615104	ALK antibody	PB	RT-PCR, *NPM*::*ALK* immunocytochemical, ALK antibody titers	28	risk of relapse: MDD positivity (more than 10 NCNs *NPM::ALK*) and antibody titer ≤ 1/2250 vs remaining patients, 62.5% vs 15% (*P* = 0.02)	MRD
ALK-positive ALCL	Mussolin, L et al. [164]	22907048	ALK antibody	PB	RT-PCR, *NPM::ALK* immunocytochemical, ALK antibody titers	128	MDD + : 59% anti-ALK response + : 96% prognosis: high risk (bHR): MDD-positive and antibody titer ≤ 1/750, 26/128 (20%), PFS 28%, OS 71% low risk (bLR): MDD negative and antibody titer > 1/750, 40/128 (31%), PFS 98%, OS 98% intermediate risk (bIR): all remaining patients, 62/128 (48%), PFS 68%, OS 83% (*P* = 0.02)	prognosis
ALK-positive ALCL	Iijima-Yamashita, Yuka et al. [165]	29030834	ALK antibody	PB	RT-PCR, real-time PCR (qPCR), *NPM::ALK* immunocytochemical, ALK antibody titers	60	Fifty-two percent were MDD + : RT-PCR 52%. 37% pts > 10NCNs 51% pts antibody titer > 1/750 PFS: high risk (HR): MDD-positive and antibody titer ≤ 1/750, 5/8, 2-year PFS 30% low risk (LR): MDD negative and antibody titer > 1/750, 0/11, 2-year PFS 100% intermediate risk (IR): all remaining patients, 1/15, 2-year PFS 93.3% *P* = 0.001	MRD prognosis

*ALCL* Anaplastic large cell lymphoma, *PB* peripheral blood, *MDD* Minimal disseminated disease

#### Application of antibodies in liquid biopsy of T-cell lymphoma

Genetic alterations in tumors lead to changes in the expression of "self-antigens," which play a critical role at the intersection of the immune system and tumor development, occurring throughout the malignant process [166]. This results in the production of autoantibodies, which can serve as biomarkers for tumor diagnosis and predicting relapse [167]. ALCL accounts for approximately 15% of pediatric non-Hodgkin lymphoma (NHL) cases [168], with the majority being associated with the t(2;5) translocation, leading to expression of the tyrosine kinase ALK. ALK influences various cellular responses, including proliferation, growth, and apoptosis. Autoantibodies against ALK are produced in ALK-positive ALCL patients [169]. Mussolin et al. [169], included 28 cases of ALK-positive ALCL and detected circulating antibodies against the ALK protein kinase in 25 out of 28 samples at diagnosis. Of the 28 patients, 15 (54%) had positive *NPM::ALK* transcripts in peripheral blood samples. They further investigated the correlation between MRD at the end of treatment and antibody titers and found a significant correlation between *NPM::ALK* transcript copy numbers and antibody titers. Patients with low antibody titers at the end of treatment experienced relapse. They defined a cutoff value for antibody titers to predict relapse and found that an antibody titer cutoff of 1/2250 had the best sensitivity (75%) and specificity (55%). Patients with antibody titers below the cutoff value had a higher relapse rate compared to children with higher antibody titers. This study provides evidence that ALK antibodies have potential diagnostic value in ALK-positive ALCL. Furthermore, risk stratification based on the levels of ALK antibody titers may serve as a prognostic marker for ALCL and evaluate its correlation with MRD status during chemotherapy.

#### Detection of antibodies

According to the different detection signals, antibody detection techniques can be categorized into optical detection, electrochemical detection, and biosensor detection, among others [170]. These techniques have their own advantages, limitations, and scopes of application, and the selection of appropriate techniques depends on specific experimental requirements and application scenarios. Among them, optical-based detection is commonly used in signal detection. Common optical antibody detection techniques include FCM, immunohistochemistry (IHC), protein microarray, and enzyme-linked immunosorbent assay (ELISA) [171–173]. However, many challenges are faced in antibody detection. For instance, the presence of non-specific components in complex biological fluids can trigger matrix effects, resulting in non-specific adsorption and false-positive results. Sensitivity and detection limits of antibodies are also crucial for early disease diagnosis, as antibody abundance in the human body is relatively low during the early stages of disease. Additionally, to improve detection efficiency, the antibody detection process needs to be rapid. For global healthcare application, the detection strategy should also be convenient and affordable. Currently, in liquid biopsies of T-cell lymphomas, antibody detection can be used to measure the expression levels of certain proteins to aid in disease diagnosis and classification. It has been found that ALK is the primary marker applied for diagnosing and predicting tumors in T-cell lymphomas using liquid biopsy, demonstrating the significance of MRD application for anaplastic large cell lymphoma. In conclusion, the selection and integration of appropriate antibody detection techniques depend on the evolving tumor antigen expression and antibody detection technologies, tailored to different detection objectives and methods.

### Cytokines

Cytokines in tissues and physiological fluids serve as potential and suitable biomarkers. Cytokines are soluble proteins with low molecular weight (approximately 6–70 kDa) that are secreted from various cells, including lymphocytes, macrophages, natural killer (NK) cells, mast cells, and stromal cells [174]. They play a role in intercellular signaling and participate in various biological processes, including cell proliferation, differentiation, and immune response. Abnormal cytokine levels lead to cytokine storms and contribute to various diseases [175, 176]. Therefore, the quantification of cytokines is valuable for disease diagnosis and treatment. Individual cytokines may be secreted by different cell types and can act on several cell types, resulting in multiple biological activities [177]. Changes in cytokine levels in various biological fluids, such as serum, blood, stool, saliva, and sweat, provide valuable information for the diagnosis, staging, and prognosis of various diseases. Cytokine levels are considered important indicators for assessing clinical conditions. Therefore, accurate quantification of cytokines provides valuable information in clinical settings to monitor the immune status of patients and adjust the treatment for different diseases [178–180]. In the context of liquid biopsies of lymphomas, cytokines are often used as biomarkers to aid in disease diagnosis or monitor treatment efficacy. Table 5 summarizes the application of cytokine detection in liquid biopsies of T-cell lymphomas.

**Table 5:** Clinical application of cytokines T cell lymphoma

Lymphoma subtypes	Author	PMID/doi	Biomarker	Liquid Biopsy	Method	No. of patients	Results	Clinical applications
ENKTL	Heemann, Christina et al. [144]	22573350	cytokines, sTNFRII	PB	ELISA, sTNFRI, sTNFRII, IL-10, and sIL-4R	117	sTNFRII serum levels ≥ 2.16 ng/mL had a 2.07-fold increased relative risk for shorter overall survival (OS; univariate: *P* = 0.0034; multivariate: HR, 2.07; CI, 0.92–4.70 with *P* = 0.081) and a 2.49-fold higher risk for shorter EFS (univariate: *P* = 0.00068; multivariate: HR, 2.49; CI, 1.22–5.08 with *P* = 0.012)	prognosis
ENKTL	Ni, Mingli et al. [181]	30643796	cytokine, IL-13	PB	ELISA	15	IL-13 inhibited chemotherapy sensitivity of NK/T-cell lymphoma cells by promoting ABCC4 expression	drug resistance
ENKTL	Huo, Jia et al. [182]	35226228	cytokine, IL-10	PB	ELISA	50	IL-10 contributes to the resistance of ENKTL cells by promoting ABCC4 expression	drug resistance

*ENKTL* extranodal NK/T-cell lymphoma

#### Application of cytokines in liquid biopsy of T-cell lymphoma

Previous research has indicated a link between the presence cytokines or their receptors in liquid biopsies an clinical outcomes in lymphoma [183, 184]. Heemann et al. investigated the serum levels of sTNFRI, sTNFRII, IL-10, and sIL-4R in 117 patients with peripheral T-cell NHL (T-NHL) using ELISA. They found that patients with serum levels of sTNFRII ≥ 2.16 ng/mL had decreased OS and event-free survival, suggesting that cytokines detected through liquid biopsy play a crucial role in predicting clinical outcomes in patients with peripheral T-NHL [144].

NK/T-cell lymphoma is characterized by high relapse rates and poor survival outcomes, even after chemotherapy or radical radiotherapy, mostly due to chemotherapy resistance. Cytokines play a critical role in drug resistance. Ni et al. found that interleukin-13 (IL-13) and ABCC4 were highly expressed in NK/T-cell lymphomas [185]. IL-13 promoted the expression of ABCC4, leading to increased resistance of NK/T-cell lymphoma cells to asparaginase. Huo et al. demonstrated that IL-10 enhanced chemotherapy resistance of lymphoma cells to gemcitabine by promoting ABCC4 expression [186]. Inhibition of ABCC4 may effectively improve the treatment of drug-resistant NK/T-cell lymphoma. Hence, dynamic monitoring of cytokines in bodily fluids provides valuable insights into mechanisms of medication resistance, hence guiding the development of innovative therapeutic strategies for this aggressive disease.

#### Detection of cytokines

Accurate detection of cytokines in practice poses challenges due to their characteristics, such as low concentration levels (in the picomolar range), dynamic secretion, short half-life, and complex network relationships [187, 188]. Several methods are available for cytokine detection in blood, with two common quantitative methods being ELISA [189] and PCR [190]. These methods are reliable but time-consuming, requiring expensive laboratory equipment, trained professionals, lengthy sample preparation (over 6 h), and complex sample handling procedures. Additionally, some methods are unable to measure multiple cytokines in real-time [174].

High-throughput testing methods, such as Luminex, are increasingly applied for rapid cytokine detection. Luminex is a multiplex immunoassay capable of simultaneously detecting various cytokines using fluorescent microbeads, each tagged with specific antibodies to recognize and bind different cytokines. The fluorescence intensity of each microbead is then measured using a fluorescence detector [191]. However, compared to conventional detection techniques such as PCR, the technical complexity and high costs, including equipment and testing costs, limit its widespread application.

FCM is another high-throughput single-cell analysis technique that can detect different types of cells and multiple cytokines in blood. FCM instruments isolate individual cells from blood samples and can simultaneously detect multiple cytokines, providing significant advantages over other single-cell methods [192, 193]. However, similar to Luminex, technical complexity and cost are major limitations of FCM. Therefore, there is potential for the development of more affordable devices based on the principles of FCM.

In recent years, mass cytometry, or cytometry by time-of-flight, has gained increasing attention for rapid single-cell analysis. This technique combines the capabilities of FCM and mass spectrometry, allowing for the simultaneous measurement of over 40 cellular parameters at single-cell resolution. It significantly enhances the high-dimensional quantitative analysis of the impact of bioactive molecules on cell populations, thus advancing the evaluation of complex cellular systems [194, 195]. Its limitations include the need for individual probes with distinct metal isotopes and limited cell throughput, necessitating a higher number of cells as input [196]. Biosensors, which integrate nanotechnology, are gaining attention for their potential in real-time cytokine monitoring and widespread application. These devices combine biological components with physical and chemical detectors, demonstrating significant sensing potential [188].

It's important to note that the mentioned methods and technologies have been widely applied in cytokine detection, each with its own advantages and limitations. The choice of method should consider specific research objectives, experimental requirements, and feasibility [197–200].

## Ongoing clinical trials of liquid biopsy for T-cell lymphoma

Several clinical trials are currently investigating cfDNA/ctDNA, CTCs, cytokines, quantitative EBV DNA, antibodies, and some also involve epigenetic DNA methylation and T-cell-specific neoantigens. Most studies focus on single biomarkers, while a few studies explore comprehensive biomarker profiling using multiple dimensions. The clinical applications of these biomarkers include a diverse range of areas, including treatment response assessment, MRD status evaluation, risk assessment for relapse, dynamic monitoring, guidance for treatment decisions, investigation of drug resistance and tumor clonal evolution, correlation with patient survival, predicting treatment efficacy and prognosis, assessment of tumor burden, among other applications. The design of these studies highlights the extensive coverage of liquid biopsies in diagnosis and treatment of T-cell lymphoma. Therefore, there is a broad scope for the future application of liquid biopsies in T-cell lymphoma (Table 6).

**Table 6:** Ongoing Clinical Trial of Liquid Biopsy for T-cell Lymphoma

NCT/ChiCTR Number	Biomarker	Liquid Biopsy	Disease	Study Title
NCT03829540	cytokine	serum	T-cell Lymphoma|T-cell Leukemia	CD4CAR for CD4 + Leukemia and Lymphoma
NCT03789617	cytokine	plasma	EBV Associated Extranodal NK/T-cell Lymphoma|EBV-Associated GastricCarcinoma or Esophageal AdenoCarcinoma	A Multi-center, Single-arm, Open, Phase I/IIa Clinical Trial to Evaluate the Efficacy and Safety of EBViNT Cell (EBV Specific Autologous CD8 + T Cell) in Patients With Treatment Failed Epstein Barr Virus (EBV)-Positive
NCT05398614	cytokine	peripheral blood	T-ALL|Lymphoma	SENL101 Autologous T Cell Injection in Adults With Relapsed or Refractory CD7 + Hematolymphoid Malignancies
NCT04848064	cytokine	peripheral blood	Adult T-Cell Leukemia/Lymphoma/ Primary Cutaneous T-Cell Non-Hodgkin Lymphoma	Third-Party Natural Killer Cells and Mogamulizumab for the Treatment of Relapsed or Refractory Cutaneous T-cell Lymphomas or Adult T-Cell Leukemia/Lymphoma
NCT04676789	EBV-DNA	peripheral blood	Extranodal NK/T-cell Lymphoma, Nasal Type	Anti-PD-1 Antibody and Pegaspargase Combined With Radiotherapy in Early-Stage ENKTL
NCT05833893	ctDNA and EBV copy number	peripheral blood	NK/T-cell Lymphoma|Newly Diagnosed|Advanced Lymphoma	Clinical Study of XPO1 Inhibitor Selinexor Combined With COPL in Newly Diagnosed Advanced NK/T-cell Lymphoma
NCT05885464	MRD	peripheral blood	/Lymphoma|Lymphoblastic Leukemia	A Study Evaluating the Safety and Efficacy of BEAM-201 in Relapsed/Refractory T-Cell Acute Lymphoblastic Leukemia (T-ALL) or T-Cell Lymphoblastic Lymphoma (T-LL)
NCT05745714	MRD	peripheral blood	Acute Lymphoblastic Leukemia	HEM-iSMART-C: Ruxolitinib + Venetoclax + Dexamethasone + Cyclophosphamide and Cytarabine in Pediatric Patients With Relapsed or Refractory Hematological Malignancies
NCT05740449	MRD	peripheral blood	Acute Lymphoblastic Leukemia	HEM-iSMART-A: Decitabine / Venetoclax and Navitoclax in Pediatric Patients With Relapsed or Refractory Hematological Malignancies
NCT05658640	MRD	peripheral blood	Acute Lymphoblastic Leukemia	HEM iSMART-D: Trametinib + Dexamethasone + Chemotherapy in Children With Relapsed or Refractory Hematological Malignancies
NCT05626400	MRD	peripheral blood	T-cell Acute Lymphoblastic Leukemia/Lymphoma	Clinical Study of Senl-T7 CAR T Cells in the Treatment of Relapsed and Refractory CD7 + Acute T-ALL/T-LBL
NCT05618041	MRD	peripheral blood	Acute Lymphoblastic Leukemia|Lymphoma|Multiple Myeloma	The Safety and Efficay Investigation of CAR-T Cell Therapy for Patients With Hematological Malignancies
NCT05212584	MRD	peripheral blood	Relapsed/Refractory, High Risk Hematologic Malignancies|T-ALL/Lymphoma	CD7 CAR-T Cell Treatment of Relapsed/Refractory CD7 + T -Acute Lymphoblastic Leukemia/ Lymphoma
NCT05043571	MRD(Ig/TCR)	peripheral blood	Lymphoblastic Leukemia, Acute, Childhood|Lymphoblastic Leukemia|Lymphoblastic Leukemia	CARTALL: Chimeric-Antigen Receptor (CAR) T-Cell Therapy for Relapsed/ Refractory T-Lineage Acute Lymphoblastic Leukaemia
NCT04315324	MRD	peripheral blood	Recurrent T Acute Lymphoblastic Leukemia|Refractory T Acute Lymphoblastic Leukemia	Study to Test AKR1C3-Activated Prodrug OBI-3424 (OBI-3424) in Patients With Relapsed/Refractory T-Cell Acute Lymphoblastic Leukemia (T-ALL)
NCT03297697	MRD(Ig/TCR)	peripheral blood	Peripheral T Cell Lymphoma	Minimal Residual Disease in Peripheral T-cell Lymphoma
NCT03117751	MRD	bone marrow, blood, and cerebrospinal fluid	Acute Lymphoblastic Leukemia|Acute Lymphoblastic Lymphoma	Total Therapy XVII for Newly Diagnosed Patients With Acute Lymphoblastic Leukemia and Lymphoma
NCT02737046	MRD(TCR)	peripheral blood	Adult T-cell Leukemia-Lymphoma|ATLL	Belinostat Therapy With Zidovudine for Adult T-Cell Leukemia-Lymphoma
NCT04480125	cfDNA	peripheral blood	Peripheral T-cell Lymphoma	Azacitidine Combined With Chidamide in the Treatment of Newly Diagnosed PTCL Unfit for Conventional Chemotherapy
NCT04480099	cfDNA	peripheral blood	Peripheral T-cell Lymphoma	Targeted Drug Combined With CHOP in the Treatment of Newly Diagnosed Peripheral T-cell Lymphoma
ChiCTR2200060450	ctDNA	peripheral blood	Peripheral T-cell lymphoma (PTCL)	Dynamic monitoring of peripheral T-cell lymphoma (PTCL) patients in China based on circulating tumor DNA
NCT05230680	MRD	peripheral blood	T Cell Lymphoma	Azacitidine-CHOP for Patients With Nodal T-cell Lymphoma With T-follicular Helper Phenotype (ACANTUS)
NCT05149170	MRD	peripheral blood	Early-stage|Extranodal NK-T-Cell Lymphoma, Nasal and Nasal-Type	Radiotherapy and Anti-PD-1 in Low-risk ES-ENKTCL
NCT04423536	SNP Signature	Liquid Biopsy	Natural Killer T-cell Lymphoma	Predictive Value of a SNP Signature and Liquid Biopsy in Natural Killer T-cell Lymphoma
NCT04881838	NPM-ALK or ALK-variants level	peripheral blood or bone marrow	Pediatric Anaplastic Large Cell Lymphoma	CCCG-ALCL-2020 for Chinese Children and Adolescents With Newly Diagnosed High-risk ALCL
NCT04904146	Epigenetic changes	peripheral blood	Mycosis Fungoides|Sezary Syndrome	Predictive and Prognostic Biomarkers in Patients With Mycosis Fungoides and Szary Syndrome
NCT05157581	MRD	peripheral blood	Sezary Syndrome	Extracorporeal Photopheresis in Sezary Syndrome
NCT04582487	genomic, DSRP, phosphoproteomic	bone marrow and/or peripheral blood	T Acute Lymphoblastic Leukemia|Early T Acute Lymphoblastic Leukemia|T-lymphoblastic Lymphoma|Etp All	Advancing Chemical and Genomic Strategies for Relapsed/Refractory T-ALL and ETP-ALL
NCT03385226	mononuclear cell phenotyping, T cell clones, tumour infiltrating lymphocyte specific neo-antigens	peripheral blood	Cutaneous T Cell Lymphoma|Mycosis Fungoides/Sezary Syndrome	A Trial Assessing the Effect of Pembrolizumab Combined With Radiotherapy in Patients With Relapsed, Refractory, Specified Stages of Cutaneous T-cell Lymphoma (CTCL) Mycosis Fungoides (MF)/Sezary Syndrome (SS)
NCT04676087	Cytokines,T cell populations( T-regulatory cells and CD8 + cytotoxic T-cells),Changes in genomic profiles	peripheral blood	Mycosis Fungoides|Primary Cutaneous T-Cell Non-Hodgkin Lymphoma|Sezary Syndrome	Mogamulizumab and Extracorporeal Photopheresis for the Treatment of Sezary Syndrome or Mycosis Fungoides

## Challenges of liquid biopsy

Before liquid biopsy technology can be clinically promoted for T-cell lymphoma, the following critical issues need to be addressed.

Standardization of biomarker detection: Liquid biopsy involves the detection of various biomarkers such as cfDNA/ctDNA, CTCs, and EBV DNA. The detection methods for these biomarkers need standardization, clinical implementation, and validation. For example, sufficient sensitivity is required to detect low concentrations of cfDNA/ctDNA and EBV DNA [201]. Although NGS technology shows enhanced detection sensitivity, it generates issues such as incomplete information and false positives [156, 202]. For example, cfDNA may be confounded by clonal hematopoietic mutations of derived uncertain potential (CHIP) [203]. To address these issues, researchers have proposed methods such as high-depth leukocyte paired sequencing for classifying and quantifying the sources of cfDNA variants [204].

Applications in diagnosis: In liquid biopsy, it is crucial to determine tumor origin and establish diagnostic thresholds [205–208]. For example, a systematic analysis of cfDNA methylation profiles can be used to trace the origin of tumor tissue [209]. Additionally, validating the effectiveness, accuracy, and practicality of liquid biopsy diagnostics is also essential.

Applications in prognostic or treatment monitoring: Determining the optimal cut-off value for prognosis and liquid biopsy criteria for treatment response is an issue that needs to be addressed [210, 211]. Yukako Shiomi-Mouri utilized the Cell Search™ system to precisely quantify CTCs in breast cancer patients, demonstrating substantial prognostic significance at a CTC threshold of one [212]. Nevertheless, accurately discerning between genuine disease progression and apparent progression in patients undergoing post-treatment monitoring still poses considerable challenges. Research has shown that utilizing longitudinal ctDNA profiling can enable early differentiation between pseudoprogression and true progression [213].

Evaluation of sampling frequency and endpoints: Standardizing specific frequencies for continuous fluid sampling and determining sampling endpoints are needed in treatment monitoring [214]. Additionally, continuous patient sampling in liquid biopsy requires ethical regulation and management.

Validation of the clinical application of liquid biopsy in T-cell lymphoma: Compared to other solid tumors, the application of liquid biopsy in T-cell lymphoma is relatively limited. Therefore, large-scale clinical research is needed to validate its clinical application in this context.

Addressing these issues requires interdisciplinary collaboration and large-scale clinical research to ensure the accuracy, reliability, and clinical feasibility of liquid biopsy technology in T-cell lymphoma.

## Recommendations and outlook of liquid biopsy in T-cell lymphoma

T-cell lymphoma is a group of malignant tumors with strong invasiveness and extremely complex heterogeneity. As a non-invasive detection method, liquid biopsy has emerged as a promising new modality for T-cell lymphoma in early diagnosis, postoperative monitoring, treatment response, and tumor drug resistance. The method provides tumor molecular information and overcomes tumor heterogeneity, allowing real-time monitoring of tumor progression and personalized treatment. Due to the fact that different subtypes of T-cell lymphoma often present different molecular events and disease characteristics, it is necessary to comprehensively consider various factors such as tumor subtypes, detection targets, the complexity of detection technology, detection cycle, and cost in real clinical scenarios when making diagnostic and treatment decisions. Unfortunately, there are currently no established guidelines or recommended diagnostic and therapeutic norms based on complete evidence-based medicine from literature and research findings in the field. In this work, we referred to the latest World Health Organization classification standards and made recommendations around the pathological characteristics and key molecular events of the main subtypes of T-cell lymphoma, taking into account the characteristics of different liquid biopsy techniques (Table 7).

**Table 7:** Recommended liquid biopsy detection assays for main T-cell lymphoma subtypes concerning pathological and genetic features

Subtype	Pathological characteristics	Genetic alternations	Recommended LB assays
nTFHL-AI	1. Pan T-cell antigens: CD2, CD3, and CD5; 2. TFH cell-associated markers, such as PD1, ICOS, CXCL13, CD10, and BCL6; 3. EBV-positive B cells are nearly always present	*IDH2*^*R172*^*, RHOA*^*G17V*^*, TET2, DNMT3A*, T-cell receptor (TCR) signaling and activation (eg, RHOA, VAV1, CD28, ICOS, FYN, LCK), TCR clonal rearrangements (most cases)	1.cfDNA/ctDNA (★★★) 2. CTCs (★★) 3. EBV DNA (★)
ALK-positive ALCL	CD30 + ; ALK + ; EMA + ; CD25 + ; cytotoxic granules + /–, CD4 + /–; CD3–/ + ; CD43 +	*ALK* rearrangement, *NOTCH1, TP53, EP300, KMT2D, TCR* clonal rearrangements (Approximately 90% of ALK-positive ALCLs)	1. Antibodies (★★★) 2. CTCs (★★) 3.cfDNA/ctDNA (★★)
ALK-negtive ALCL	CD30 + ; EMA + ; CD25 + ; cytotoxic granules + /–; CD4 + /–; CD3–/ + ; CD43 + ; PAX5/BSAP–	*DUSP22* and *TP63* rearrangement, *TP53* loss, *JAK1, JAK3, STAT3*, TCR clonal rearrangements (most cases)	1. CTCs (★★) 2.cfDNA/ctDNA (★★)
PTCL, NOS	CD4 > CD8; antigen loss frequent (CD7, CD5, CD4/CD8, CD52); GATA3–/ + ; TBX21–/ + ; cytotoxic granules–/ + ; CD30-/ + ; CD56–/ + ; rare cases EBV +	**PTCL-GATA3**: *TP53, CDKN2A/B, PRDM1, RB1, PTEN, STAT3, MYC*; **PTCL-TBX21**: low genomic complexity and few recurrent specific genetic changes; TCR clonal rearrangements (most cases)	1.cfDNA/ctDNA (★★) 2. CTCs (★) 3. EBV DNA (★)
ENKTL	Lymphoma of NK-cell or T-cell lineage, has a very strong association with EBV	**ENKTL**: *TP53, DDX3X, STAT3, JAK3, STAT5B, TET2, KMT2D, KMT2C*; **Nodal EBV-positive T and NK-cell lymphoma**: TET2; TCR clonal rearrangements (10%-40% cases)	1. EBV DNA (★★) 2.cfDNA/ctDNA (★)

*nTFHL-AI* Nodal T-follicular helper cell lymphoma, angioimmunoblastic-type, *ALCL* Anaplastic large cell lymphoma, *ALK*-negtive ALCL, *PTCL,NOS* peripheral T-cell lymphoma, not otherwise specified, *ENKTL* extranodal NK/T-cell lymphoma, *T-LBL* T-cell lymphoblastic lymphoma, *cfDNA* circulating cell-free DNA, *ctDNA* circulating tumor DNA, *CTCs* circulating tumor cells, *MRD* Minimal residual disease, *EBV* Epstein-Barr virus, *EBV DNA* Epstein-Barr virus DNA

★★★ Prior; ★★ Acceptable; ★ Certain situations

In the World Health Organization Hematology (WHO-HAEM5) classification, nTFHL encompass a triad of nodal T-cell lymphoma classifications distinguished by the phenotype and genetic expression profiles characteristic of T-follicular helper cells. These classifications include angioimmunoblastic, follicular, and not otherwise specified types. Each expresses an array of TFH markers, including *PD1*, *ICOS*, *CXCL13*, *CD10*, and *BCL6* [215]. Furthermore, these lymphomas exhibit a congruent mutational spectrum, notably featuring loss-of-function mutations in genes associated with methylation, such as *TET2*, identified in approximately 80% of instances, and *DNMT3A*, observed in 30–40% of cases. Additional recurrent mutations involve *CD28*, *RHOA (G17V)*, and *IDH2 (R172)*, predominantly in a subset of nTFHL-AI, as well as alterations in the TCR signaling pathway [216]. TCR genes demonstrate clonal rearrangements in the majority of cases, with nTFHL-AI showing a 75%–90% occurrence rate. Moreover, clonal IG gene rearrangements, found in about 25–30% of cases, are associated with an expansion of EBV-positive B cells [217]. This subtype is characterized by a distinct immunophenotype and a well-delineated mutational profile. In light of our above research summary, we advocate for the utilization of cfDNA/ctDNA and CTCs as preferred methodologies for liquid biopsy detection. Additionally, cfDNA/ctDNA may present enhanced sensitivity for this purpose.

ALCL is a mature T-cell lymphoma typified by heterogeneous tumor cells that uniformly exhibit robust CD30 expression, frequently accompanied by compromised expression of T-cell lineage markers. ALK-positive ALCL is distinguished from ALK-negtive ALCL by its unique pathogenesis [218], which is characterized by the presence of ALK gene fusions that encode oncogenic proteins. These fusions are predominantly detected through IHC. The most common ALK rearrangement involves t(2;5)(p23;q35), which results in the fusion of nucleophosmin (*NPM1*) with *ALK*, producing a fusion protein. Additionally, recurrent mutations in *NOTCH1*, *TP53*, and epigenetic modulators such as *EP300* and *KMT2D/C* have been documented [219, 220]. Notably, the characteristic *ALK* fusions facilitate the detection of this subcategory using peripheral blood analysis for *ALK* transcripts, which offers both diagnostic and prognostic utility, rendering antibodies as the optimal assay for the liquid biopsy of ALK-positive ALCL. Moreover, numerous studies have corroborated that specific immunophenotypic and mutational profiles render CTCs and cfDNA/ctDNA as exceptionally promising modalities for liquid biopsy applications.

ALK-negative ALCL is recognized as a heterogeneous entity. ALK-negative ALCL with *TP63* rearrangements, *TP53* loss, and heightened expression of IL-2Rα correlate with adverse prognoses [215]. Original studies indicated that *DUSP22* rearrangement was linked to an encouraging five-year OS rate akin to that of ALK-positive ALCL, yet subsequent research has failed to uphold this finding [221]. Approximately 60% of ALK-negative cases demonstrate JAK-STAT signaling pathway activation, predominantly through mutations in *JAK1*, *JAK3*, and *STAT3*, or through rearrangements involving *TYK2*, *ROS1*, and *FRK* [222]. In essence, this subtype is characterized by the absence of *ALK* fusion genes and the presence of *CD30*, which sets it apart from other subtypes and is usually accompanied by genetic alterations in the JAK-STAT signaling pathway. Consequently, CTCs and cfDNA/ctDNA may present more suitable options for liquid biopsy assays.

In the WHO-HAEM5 classification, the category of PTCL, NOS is recognized as a heterogeneous category and is identified through a process of exclusion, with a discerning differential diagnosis that notably encompasses nTFHL, among other entities [215]. Two distinct biological subtypes of PTCL, NOS, termed PTCL-TBX21 and PTCL-GATA3, have been delineated based on their respective transcriptional profiles, which mirror those of T-helper type 1 and T-helper type 2 cells [223]. PTCL-GATA3 is characterized by frequent deletions and mutations in the *TP53* and *PRDM1* genes, loss of *CDKN2A/B*, *RB1*, and *PTEN*, as well as amplifications of *STAT3* and *MYC*, whereas PTCL-TBX21 is more heterogeneous, potentially encompassing a subset with a cytotoxic gene expression signature and a propensity for aggressive clinical behavior. PTCL-TBX21, in contrast, exhibits low genomic complexity and a scarcity of recurrent, specific genetic alterations, such as gain of chromosome 5 and a focal gain at 14q32 that includes the *BCL11B* gene [224]. PTCL, NOS has been incorporated into multiple investigations involving cfDNA/ctDNA and CTCs, as previously mentioned. The outcomes from these studies have affirmed the utility of both liquid biopsy modalities for diagnostic purposes and MRD tracking. In light of the pathological and genetic attributes of this lymphoma variant, the incorporation of cfDNA/ctDNA and CTCs into liquid biopsy protocols is advocated.

Extranodal NK/T-cell lymphoma, previously designated as "nasal-type," will no longer carry this specifier in WHO-HAEM5, reflecting its established manifestation across a spectrum of extranodal locations [215]. A significant correlation exists between ENKTL and EBV infection, yet the intricacies of EBV's involvement remain elusive. Chromosomal deletion spanning 6q21-25 emerges as a recurrent genomic aberration, encompassing genes such as PRDM1, PTPRK, and FOXO3. Predominant mutations are observed in the JAK/STAT signaling pathway (notably *STAT3*, *JAK3*, and *STAT5B*), various tumor suppressor genes (e.g., *TP53*, *DDX3X*), and genes governing epigenetic regulation (e.g., *TET2, KMT2D, KMT2C*) [222]. Considering the pronounced link with EBV, the paucity of mutations reported in prior studies, and the prognostic implications of certain cytokines for drug resistance, EBV DNA quantification proves superior as a liquid biopsy assay. Additionally, cfDNA/ ctDNA assays and cytokine profiling may offer valuable insights in selected clinical scenarios.

There are multiple potential future directions that are emerging for liquid biopsies. This method provides tumor molecular information and overcomes tumor heterogeneity, enabling real-time monitoring of T-cell lymphoma progression and personalized patient treatment. Although it presents challenges in terms of technical and clinical implementation, liquid biopsy offers the advantage of longitudinal monitoring compared to traditional tissue biopsies. This can assist clinical oncologists in gaining a broader molecular understanding of the disease.

## Data Availability

Not applicable.

## Abbreviations

cfDNA: Circulating cell-free DNA
ctDNA: Circulating tumor DNA
CTCs: Circulating tumor cells
MRD: Minimal residual disease
FCM: Flow cytometry
EBV: Epstein-Barr virus
EBV DNA: Epstein-Barr virus DNA
ALCL: Anaplastic large cell lymphoma
PTCL: Peripheral T-cell lymphoma
ENKTL: Extranodal NK/T-cell lymphoma
nTFHL-AI: Nodal T-follicular helper cell lymphoma, angioimmunoblastic-type
PTCL, NOS: Peripheral T-cell lymphoma, not otherwise specified
T-LBL: T-cell lymphoblastic lymphoma
T-ALL: T-cell lymphoblastic leukemia
T-ALL/T-LBL: T-lymphoblastic leukaemia / lymphoma
ATLL: Adult T-cell leukemia/lymphoma
T-LGLL: T-large granular lymphocytic leukaemia
T-PLL: T-prolymphocytic leukemia
HTLV-1: Human T-lymphotropic virus 1
MF: Mycosis fungoides
SS: Sézary syndrome
CTCL: Cutaneous T-cell lymphoma
NGS: Next-generation sequencing
HSCT: Hematopoietic stem cell transplantation
GE: Genome equivalent
WES: Whole exome sequencing
WGS: Whole genome sequencing
WGBS-Seq: Whole genome bisulfite sequencing
HTS: High-throughput sequencing
AS-PCR: Allele-specific polymerase chain reaction
RQ-PCR: Real-time quantitative PCR
dPCR: Digital polymerase chain reaction
BEAMing: Beads, emulsion, amplification, and magnetics
CS-PCR: Clonal specific PCR
MFC: Multicolor flow cytometric
IHC: Immunohistochemistry
ELISA: Enzyme-linked immunosorbent assay
mpFC: Multiparameter flow cytometry
CI-Rs: Cumulative incidence of relapses
NHL: Non-Hodgkin lymphoma
OCRs: Open chromatine regions
SC/LH: Small cell/lymphohistiocytic
LCT: Large-cell transformation
TCR: T-cell receptor
PB: Peripheral blood
CSF: Cerebrospinal fluid
FNA: Fine-needle aspirate
BM: Bone marrow
OS: Overall survival
DFS: Disease-free survival
PFS: Progression-free survival
CyTOF: Mass cytometry
CHIP: Clonal hematopoietic mutations of derived uncertain potential.
LB: Liquid biopsy
